# Making the case for prophylactic use of betaine to promote brain health in young (15–24 year old) athletes at risk for concussion

**DOI:** 10.3389/fnins.2023.1214976

**Published:** 2023-09-22

**Authors:** Leena S. Knight, Thomas A. Knight

**Affiliations:** Biology Department, Whitman College, Walla Walla, WA, United States

**Keywords:** betaine, concussion, osmoregulation, excitotoxicity, neuroprotection, female, athletes

## Abstract

Betaine supplementation in the context of human nutrition, athletic performance, and clinical therapy demonstrate that the osmolyte and methyl donor, betaine, is cytoprotective and beneficial to human health. These studies also demonstrate that betaine supplementation in healthy humans is straight-forward with no reported adverse effects. Here, we explore betaine uptake in the central nervous system (CNS) and contribute to evidence that betaine may be uniquely protective to the brain. We specifically describe the therapeutic potential of betaine and explore the potential implications of betaine on inhibition mediated by GABA and glycine neurotransmission. The influence of betaine on neurophysiology complement betaine’s role as an osmolyte and metabolite and is consistent with clinical evidence of betaine-mediated improvements to cognitive function (reported in elderly populations) and its anti-convulsant properties. Betaine’s therapeutic potential in neurological disorders including epilepsy and neurodegenerative diseases combined with benefits of betaine supplementation on athletic performance support the unique application of betaine as a prophylaxis to concussion. As an example, we identify young athletes (15–24 years old), especially females, for prophylactic betaine supplementation to promote brain health and resilience in a cohort at high risk for concussion and for developing Alzheimer’s disease.

## 1. Introduction

Betaine, or trimethylglycine, is a stable, organic osmolyte that can be acquired through diet via food sources such as beets, whole wheat, shellfish, and spinach (Craig, 2004) or can be metabolically synthesized. Betaine plays an important role in water balance helping protect cells and tissues from osmotic stress by preserving fluid dynamics, biomolecular structure, ionic balance, and many other cellular properties dependent on osmotic balance. Betaine is also an essential metabolite serving as a methyl donor in the conversion of homocysteine–which can be toxic to cells–into methionine, an essential amino acid. These principal functions allow betaine to serve dual, vital roles in cellular physiology. Dietary betaine supplementation has been linked with numerous therapeutic benefits. Arumugam et al. (2021) provide a current, comprehensive review of betaine and its numerous protective benefits offering an excellent summary table of all experimental and clinical studies completed through 2020. Based on this summary and other reviews, it is clear that the benefits of betaine have been most comprehensively studied in liver and kidney physiologies where osmotic stress and homocysteine toxicity play significant roles. Favorable biological outcomes have been reported in many other organ systems, in a variety of animal models, and in human clinical therapeutic and athletic performance applications. Importantly, the literature consistently demonstrates that betaine is stable, easily administered, and has broad, significant therapeutic potential.

This review focuses on the protective benefits of betaine in the central nervous system (CNS), highlights the additional role betaine may play in inhibitory neural signaling, and considers novel applications of betaine supplementation in mitigating damage associated with concussion. Specifically, we will: (a) identify aspects of betaine metabolism that uniquely position betaine in neuroprotection; (b) review existing literature on betaine’s potential impact on inhibitory neurotransmission; and, (c) consider dietary supplementation of betaine as a prophylactic to protect nervous tissue from thermal (high temperature), osmotic, metabolic, and excitotoxic stress to mitigate injury and aid recovery.

## 2. Betaine and osmotic stress in neurophysiology

Organic brain osmolytes are especially essential to CNS function since changes in water and ion balance can dramatically affect intracranial pressure and electrochemical gradients necessary for proper neurotransmission. It is well-established that osmolytes are key to the ability for cells to respond to changes in tonicity or osmolality, to maintain cell volume, and to preserve neural signaling and overall nervous tissue function. An emerging role of osmolytes in macromolecular crowding, protein folding, and preservation of biomolecular structure particularly under conditions of osmotic, thermal, and pH, stress, adds yet another dimension to the cytoprotective properties of osmolytes in nervous tissue (Bounedjah et al., 2012; Kushwah et al., 2020). For example, hypertonicity leads to the formation of stress granules that compromise mRNA machinery and to abnormal bundling of microtubules–a key cytoskeletal structure–that damage cellular motility. Bounedjah et al. (2012) demonstrate that pre-treatment of cells with betaine protects cells from hyperosmolarity induced loss of cell functions and improves cell survival. The 2020 review by Kushwah et al. (2020) highlight the role of misfolded and/or aggregated proteins in the pathogenesis of neurodegenerative disorders such as Alzheimer’s disease (AD) and Parkinson’s disease (PD) as well as neurological sequelae associated with metabolic disorders such as type-2 diabetes.

Betaine, if not catabolized (see specify section “3. Neuroprotective properties of betaine as a methyl donor”), can serve as an organic osmolyte and has been linked with the prevention of neurodegenerative disorders such as Alzheimer’s and Parkinson’s disease (Kushwah et al., 2020; Willingham et al., 2020), possibly through the suppression of amyloid aggregates (Arora et al., 2004; Liu et al., 2014). Betaine’s anti-inflammatory properties may also be related to preventing endoplasmic reticulum stress resulting from mis-/un-folded proteins, through inhibition of caspase aggregation mediating apoptosis (Xie et al., 2020) and preventing inflammasome assembly (Zhao et al., 2018; Zhang et al., 2022). Indeed, recent data on the anti-inflammatory role of betaine has been linked with attenuation of pain (Hassanpour et al., 2020; Nobari et al., 2021a; Tiwari and Hemalatha, 2022) and with treatment of affective disorders like depression (Chen et al., 2021; Zhang et al., 2022). Therefore–in addition to regulating cell volume, fluid dynamics, etc.–betaine, as an osmolyte, plays an essential role in protein folding and aggregation that benefit CNS physiology and affect pathogenesis.

## 3. Neuroprotective properties of betaine as a methyl donor

Betaine also plays an essential role in the activated methyl cycle, serving as a methyl donor for the conversion of homocysteine (a toxin) into methionine. The metabolic role of betaine in human nutrition is multi-fold and comprehensively summarized by Craig (2004). Dietary betaine, in particular, detoxifies homocysteine while sparing methionine and choline levels and converts homocysteine into dimethylglycine, sarcosine, and glycine, all valuable metabolites. It is important to note that the transmethylation of methionine is key to DNA methylation processes in addition to regulation of homocysteine toxicity; therefore, there is increasing interest in understanding the role of betaine in the epigenetic regulation of genes. For a summary of this literature see section “5.1. Betaine in athletes”, the 2021 review by Arumugam et al. (2021) and the 2020 review by Randunu and Bertolo (2020) which discusses multi-generational epigenetic implications.

The importance of the methyl cycle and homocysteine regulation in development and function of the CNS is well known (Zeisel, 2006, 2009; Eussen et al., 2007; Lever and Slow, 2010; Arumugam et al., 2021). Homocysteine toxicity in nervous tissue has been linked with seizure activity (Freed et al., 1979; Freed, 1984) and neurodegeneration (Oulhaj et al., 2010; Smith et al., 2010; Suszynska et al., 2010; Sun et al., 2017). Betaine, specifically, has been shown to have anti-convulsant properties (Freed et al., 1979; Freed, 1985; Ghoz and Freed, 1985) and to attenuate Alzheimer-like deficits resulting from homocysteine (Chai et al., 2013; Sun et al., 2017). Furthermore, studies show reduced urinary betaine concentration associated with type-2 diabetes and cardiovascular disease and, the corollary, higher levels of serum betaine associated with fewer complications and lower risk of Type 2 diabetes, see the 2022 review by Szkudelska and Szkudelski (2022). Though it remains to be tested directly, cognitive deficits observed in patients with type-2 diabetes may be a result of homocysteine neurotoxicity/neurodegeneration since dysregulation of betaine is observed in patients with obesity and diabetes (Lever et al., 2012; McEntyre et al., 2015). Furthermore, therapeutic application of betaine in offspring following exposure to induced diabetes *in utero* demonstrated promising preservation of cognitive function (Zabrodina et al., 2016). In sum, betaine offers neuroprotection under conditions of both osmotic and metabolic stress given its dual role as an osmolyte and as a methyl donor.

## 4. Betaine in the central nervous system

Betaine’s dual protective properties are beneficial to most tissues and organ systems. These benefits have been extensively studied in hepatic and renal physiologies with evidence that betaine confers benefits to other organ systems including the cardiovascular, musculoskeletal, and gastrointestinal systems. The advantages that betaine offers in homeostatic regulation at an integrated organ-system level is undoubtedly beneficial to the nervous system. However, betaine–unlike other organic osmolytes–has the potential to also directly influence inhibitory neurotransmission (see subsections “4.1. Betaine transport and implications on GABA-mediated inhibition” and “4.2. Betaine as a source of glycine–another key inhibitory neurotransmitter”) and thereby provide unique neuroprotection from excitotoxicity. Dietary betaine is readily absorbed in the gut via non-specific porters, distributed systemically in blood plasma, and accumulated in various tissues (Slow et al., 2009). The study by Slow et al. (2009) suggests that betaine concentrations are significantly lower in brain tissue compared to other tissues and are below plasma serum levels. However, research in our lab suggests that given supplementation brain tissue has significant capacity to accumulate betaine, especially under conditions of stress and preferentially over other osmolytes (Leena S Knight et al., 2017). Though it has been established that betaine can cross the blood-brain-barrier (Kempson et al., 2014; Wang et al., 2022) and accumulate in nervous tissue (Slow et al., 2009; Knight et al., 2017), further investigation is necessary to reconcile how much betaine accumulates and its impact on brain function.

### 4.1. Betaine transport and implications on GABA-mediated inhibition

Betaine uptake into astrocytes and neurons is mediated by the betaine/GABA transporter 1 (BGT-1, aka GAT2) in the mouse–a class of GABA transporters with greater affinity for betaine compared to GABA and therefore preferentially transports betaine when it is available (Schousboe et al., 2004). Research from our lab suggests that intracellular betaine concentrations in the mouse hippocampus can exceed extracellular concentrations by 3-fold especially under conditions of osmotic stress. Furthermore, our data suggest a preferential uptake of betaine relative to other common organic osmolytes, e.g., taurine and creatine (Knight et al., 2017). In other words, nervous tissue actively–in an energy dependent manner–accumulates betaine by way of BGT-1 and therefore at the expense of GABA re-uptake. Although implications of betaine on GABA reuptake remain controversial–see Kempson et al. (2014) for a review of the ongoing debate regarding BGT-1 and implications on GABAergic signaling and excitability in the brain–recent studies continue to link betaine with neuronal signaling, excitability, behavior, and cognitive function (Ohnishi et al., 2019; Hassanpour et al., 2020; Latka et al., 2020; Arumugam et al., 2021; Chen et al., 2021; Rosas-Rodriguez and Valenzuela-Soto, 2021; Hardege et al., 2022; Ilyas et al., 2022) warranting continued consideration.

There are several implications of betaine transport on GABA signaling kinetics that should be considered in greater detail. First, GABA signaling is terminated through re-uptake and clearance of GABA from the synaptic cleft and extracellular space. Betaine, as a competitive substrate to BGT-1 transporters, thereby effectively reduces the rate for GABA re-uptake. Though it is estimated that only about 20% of GABA re-uptake is mediated by BGT-1 transporters with 80% mediated by other GABA transporters (GAT1-3), the location of BGT-1 transporters on astrocytes and the necessary conversion of GABA via the GABA-glutamate-glutamine cycle effectively reduces the overall GABA available in the neurotransmitter pool (Schousboe et al., 2004). Second, since other GABA transporters are located on neurons specifically at pre-synaptic terminals, reduced clearance of GABA via BGT-1 transporters, allows for more GABA to be recycled directly into the pre-synaptic terminal for continued inhibitory neurotransmission. Betaine would therefore decrease GABA re-uptake kinetics and result in a net increase in GABA available for neurotransmission. The net effect of betaine on GABA-mediated inhibitory signaling would be a longer half-life for GABA in the synaptic and extra-synaptic space and an increase in GABA available for continued neurotransmission. These effects, though subtle, would influence neurophysiological signaling under conditions of intense neural activity, such as during seizures or following brain injury. The slight shift to longer duration GABA signaling and more efficient GABA recycling may have significant influence on seizure activity and resultant excitotoxicity. When combined with the osmoregulation and metabolic support, betaine’s effect on inhibitory neurotransmission may offer significant neuroprotection.

### 4.2. Betaine as a source of glycine–another key inhibitory neurotransmitter

A number of studies report on the anti-convulsant properties of betaine (Freed, 1984; Schousboe et al., 2004; White et al., 2005). It is likely, however, that betaine’s anticonvulsant properties are not due solely to betaine’s influence on BGT-1 (Lehre et al., 2011). For example, work from the late 70s and early 80’s describe betaine as an anti-convulsant and focus on betaine’s role as a methyl donor in the conversion of homocysteine into methionine to regulate homocysteine toxicity (Freed et al., 1979; Freed, 1984). It should not be overlooked that in its role as a methyl donor, betaine (also known as trimethylglycine) is converted into glycine–another key inhibitory neurotransmitter in the central nervous system. Therefore, betaine may also contribute to inhibitory signaling in the CNS by increasing glycine availability, perhaps explaining betaine’s anti-convulsant efficacy for strychnine-induced seizures, given that strychnine is a potent inhibitor of glycine receptors (Freed, 1985). The potential influence of betaine on inhibition in the CNS is not mutually exclusive to the influence betaine has as an osmolyte and a methyl donor in nervous tissue. Indeed, it is likely that the many reports that correlate betaine supplementation with, for example, improved cognitive function in the elderly (Eussen et al., 2007) are likely a result of the numerous neurophysiological processes that betaine supports rather than any one.

When taken together, betaine is uniquely positioned to protect against many forms of stress in the nervous system, including osmotic, metabolic, and, we suggest, excitotoxic. The neuroprotective advantages of betaine are significantly reinforced by the broad, systemic benefits that betaine has on other organ systems such as renal, hepatic, and cardiovascular physiologies. In other words, betaine confers neuroprotective properties directly by augmenting nervous tissue function and also indirectly through the protection and optimization of the physiology of other organ systems.

## 5. Betaine–a neuroprotective prophylactic against concussion (mild traumatic brain injury)

Betaine’s capacity in the CNS to optimize fluid dynamics and biomolecular structure, to ameliorate homocysteine toxicity, and to shift the balance of net neural activity toward inhibition make it a unique candidate in the prevention of many neurological diseases. As described in the previous sections, betaine is a utilitarian metabolite, conspicuously positioned to buffer many key cellular processes in order to maintain physiological homeostasis. However, its ability to protect depends on its availability at the onset of physiological stress. Once nervous tissue has been damaged and neurons have been lost, replacement or recovery of post-mitotic neurons and complex nervous tissue architecture is fraught with challenges. Based on the mechanisms of action we describe above, we argue that betaine is unlikely to play a significant role in the replacement of damaged tissues though betaine has been used as a *post hoc* therapeutic aid. Instead, we suggest that betaine is uniquely positioned to promote resilience, to limit damage, and to speed recovery in nervous tissue. Therefore, betaine would need to be available prior to experiencing brain injury, and regular, daily supplementation would be most beneficial.

Given that betaine supplements are inexpensive, readily available, easily administered, and already established for human use with FDA approval in both clinical therapeutic and athletic performance settings (Craig, 2004), we propose that dietary betaine supplementation be considered a prophylaxis to concussion in young (15–24 year old) athletes, particularly in female athletes. In section “5.2. Concussion in young (15–24 year old) athletes” below we provide our detailed rationale for focusing on young, female athletes, but briefly outline the primary reasons for identifying the 15–24 year old age range. First, young adults are vulnerable to long-term sequelae following head injury given the ongoing development of the prefrontal cortex. Second, head injury during the formative years of education (high school/university) is likely to disrupt schooling and have long-term impacts on intellectual development, professional development, employment, and financial security (Sariaslan et al., 2016). Third, young adults are likely to engage in risky behavior and have limited access to support (e.g., healthcare and health insurance) for prevention, diagnosis, and treatment for head injury.

### 5.1. Betaine in athletes

In addition to clinical therapeutic settings, betaine supplementation has been used in athletic performance studies to explore the impact of betaine on exercise physiology. These studies report a variety of benefits and improved athletic performance, including improved anaerobic and aerobic metabolism, athletic performance, muscle endurance, and body composition (Trepanowski et al., 2011; Cholewa et al., 2013, 2014; Nobari et al., 2021b; Arazi et al., 2022; Machek et al., 2022) in numerous individual and team sports, e.g., running, cycling, soccer. Consistent with clinical research, these athletic performance studies do not report negative or adverse consequences to betaine supplementation. Though there are only two studies that include female athletes (Pryor et al., 2012; Cholewa et al., 2018), both report benefits of betaine supplementation and report no negative impact. Young athletes are not only at risk of dehydration and heat stress but they are also at risk of concussion (mTBI) (Harmon et al., 2013; Putukian et al., 2019; Chun et al., 2021; Pierpoint and Collins, 2021) that, in turn, puts them at higher risk for developing epilepsy, dementia, and neurodegenerative disorders like Alzheimer’s and Parkinson’s disease (Wilson et al., 2017).

### 5.2. Concussion in young (15–24 year old) athletes

Concussion (equated with mild traumatic brain injury, mTBI) is a brain injury that occurs from impact to the head or body that results in biomechanical and physiological stress to the brain (McCrory et al., 2013). Concussed patients frequently suffer from short-term, non-specific neurological symptoms, including headache, fatigue, balance, irritability, photosensitivity, confusion, and difficulty with memory, speech, and/or sleep (Dashnaw et al., 2012; McCrory et al., 2013). In their comprehensive long-term outcomes study with over 1.1 million individuals, Sariaslan et al. (2016) suggest that a single traumatic brain injury when 20–24 and 15–19 years old places individuals at the highest and second-highest risk, respectively, of adverse health and social outcomes.

Many studies of concussion have focused on sport-related concussion (SRC), estimated at 3.8 million per year in the US alone and comprising 20% of total annual concussions in the US based on a now dated study (Langlois et al., 2006). Focusing on SRC as a proxy for all concussions, females have greater risk of concussion, greater number of symptoms, greater severity of symptoms, and greater duration/prolongation of recovery from symptoms (Dick, 2009; Covassin et al., 2018; Moore et al., 2018; Bretzin et al., 2021, 2022). In their 2018 review, Covassin et al. (2018) report that the majority of research in SRC identify sex differences with female athletes experiencing more neurocognitive impairments and longer recovery times than male athletes. These include deficits in executive function, memory, and processing speed based on neurocognitive tests (Moore et al., 2018).

While many reports have tried to determine why females are at greater risk for mTBI and its effects, the causes for this remain unclear. However, there are differences between the sexes with regard to betaine. First, lower betaine content per unit tissue was observed in most tissues in female rats (Slow et al., 2009). Second, the role of the menstrual cycle has been proposed as a cause for sex-based differences in human mTBI, and indeed, betaine concentrations in female rats show much greater variability compared to males suggesting metabolic fluctuations associated with menstrual hormonal changes (Slow et al., 2009). Taken together, these observations suggest that betaine supplementation might be most beneficial to young, female athletes in supporting athletic performance and neuroprotection against the osmotic, metabolic, and excitotoxic stress that underlies mTBI.

### 5.3. Betaine as a prophylaxis to concussion

There are a striking number of points in the “neurometabolic cascade of concussion” (Giza and Hovda, 2001, 2014) where the availability of betaine might attenuate disruption. Betaine would play an osmoregulatory role (Knight et al., 2017) that may aid in osmolality and tonicity changes observed post-concussion (Kawamata et al., 2007; Knight et al., 2017; Khatibi et al., 2019; Kushwah et al., 2020) and protect against inflammatory (Di Battista et al., 2019; Kattan et al., 2023), thermoregulatory (Pegoli et al., 2020), and cerebrovascular disruptions (Kenney et al., 2016) associated with concussion and traumatic brain injury. Indeed, existing literature identifies betaine as anti-inflammatory (Zhao et al., 2018; Zhang et al., 2022), and links betaine with improved thermoregulation (Willingham et al., 2020) and protection of microvasculature (van der Vaart et al., 2023). As a methyl donor, betaine may also protect against metabolic disruption of the neurometabolic cascade, and particularly with homocysteine toxicity observed following brain injury (Schwab et al., 2002; Ueland et al., 2005; Lauretta et al., 2022). Finally, betaine has already been used clinically to improve cognitive function in neurodegenerative disorders, including dementia, Alzheimer’s, and Parkinson’s (Eussen et al., 2007; Ilyas et al., 2022). In animal models, betaine has recently been associated with various other neuroprotective roles such as in post-traumatic syringomyelia (Pukale et al., 2021), in neuropathic pain management (Hassanpour et al., 2020; Tiwari and Hemalatha, 2022), and in affective disorders (Qu et al., 2020; Chen et al., 2021; Haramipour et al., 2021; Jeyhoonabadi et al., 2022; Zhang et al., 2022).

To be clear, betaine is unlikely to serve as a stand-alone remedy for the treatment of concussion and it is unlikely to entirely prevent brain injury. However, betaine’s utility across many essential functions in nervous tissue make it an ideal candidate as a prophylactic neuroprotectant against the neurometabolic forms of stress associated with mTBI. These neuroprotective advantages are likely to be best obtained when betaine is readily available at supplemented concentrations within the body at the time of injury/cellular stress in order to reduce acute injury and improve recovery. Given that betaine is inexpensive, widely available, and easily administered (in the diet), the supplementation of betaine, especially in the diet of young female athletes as a neuroprotectant should be directly investigated–something which young athletes, parents, and researchers may agree is a “no brainer.”^1^

## Author contributions

LK wrote the first draft. Both authors contributed equally to conception and focus of this review and approved the submitted version.

## Footnotes

1 A contemporary phrase suggesting something that requires little thought to understand and is easy to accept, https://dictionary.cambridge.org/us/dictionary/english/no-brainer.
